# Prolapse into the bile duct and expansive growth is characteristic behavior of mucinous cystic neoplasm of the liver: report of two cases and review of the literature

**DOI:** 10.1007/s12328-015-0569-8

**Published:** 2015-05-08

**Authors:** Yuichi Takano, Masatsugu Nagahama, Eiichi Yamamura, Naotaka Maruoka, Hiroki Mizukami, Jun-ichi Tanaka, Nobuyuki Ohike, Hiroshi Takahashi

**Affiliations:** Division of Gastroenterology, Department of Internal Medicine, Showa University Fujigaoka Hospital, 1-30 Fujigaoka, Aoba-ku, Yokohama-shi, Yokohama, Kanagawa 227-8501 Japan; Department of Surgery, Showa University Fujigaoka Hospital, Yokohama, Kanagawa Japan; Department of Diagnostic Pathology, Showa University Fujigaoka Hospital, Yokohama, Kanagawa Japan

**Keywords:** Mucinous cystic neoplasm of the liver, Biliary cystadenoma, Ovarian-like stroma, ERCP

## Abstract

Mucinous cystic neoplasm of the liver (MCN-L) is a very rare tumor whose detailed behavior is still unknown. We describe two cases of MCN-L that exhibited extremely interesting growth patterns, and discuss the characteristics of MCN-Ls. Both cases exhibited MCN-L that originated from the left hepatic lobe (Segment 4) and then prolapsed into the left hepatic duct and common bile duct, resulting in obstructive jaundice due to expansive growth. Endoscopic retrograde cholangiopancreatographies showed the characteristic oval-shaped filling defects in the bile ducts. Endoscopic ultrasound and intraductal ultrasound were useful for differentiating the tumors from stones, since multiple septal formations were observed inside the tumors. A literature search revealed that, over the past 10 years, 15 cases of MCN-L (biliary cystadenomas with ovarian-like stroma) that showed expansive growth in the bile duct had been reported. Prolapse into the bile duct and expansive growth appear to be characteristic behavior of MCN-L. In the future, additional data on more cases needs to be collected to further elucidate MCN-L pathophysiology.

## Introduction

Mucinous cystic neoplasm of the liver (MCN-L) is an extremely rare cyst-forming epithelial tumor. This unique neoplasm is commonly observed in middle-aged women and is usually characterized by having no communication with the bile duct and rarely becoming malignant. Pathologically, the cyst wall is composed of mucin-producing cuboidal or columnar epithelium and is accompanied by ovarian-like stroma (OLS). However, detailed information on the behavior of MCN-L is lacking due to its rarity.

Along with a review of the literature, we describe two cases of MCN-L that exhibited extremely interesting growth patterns, and consider the behavior of this tumor.

## Case presentation

### Case 1

A 57-year-old woman was admitted to the emergency room complaining of sudden abdominal pain and fever. Her vital signs were blood pressure 120/76 mmHg, heart rate 102 beats/min, and body temperature 38.2 °C. Tenderness was noted from the epigastrium to the hypochondriac region, but neither muscular defense nor rebound tenderness was observed. Laboratory tests revealed elevated levels of hepato-biliary enzymes (T-bil: 2.7 mg/dL; GOT: 299 U/L; GPT: 535 U/L; ALP: 2459 U/L; γ-GTP: 2054 U/L). Cancer antigen (CA) 19-9 levels were mildly increased (99.0 U/mL, normal = 0−37 U/mL) and carcinoembryonic antigen (CEA) levels were normal (≦0.5 ng/mL, normal = 0−0.5 ng/mL). Both the hepatitis B surface antigen (HBsAg) and the hepatitis C virus antibody (HCVAb) tests were negative, and the patient had no history of excessive alcohol consumption.

**Fig. 1:**
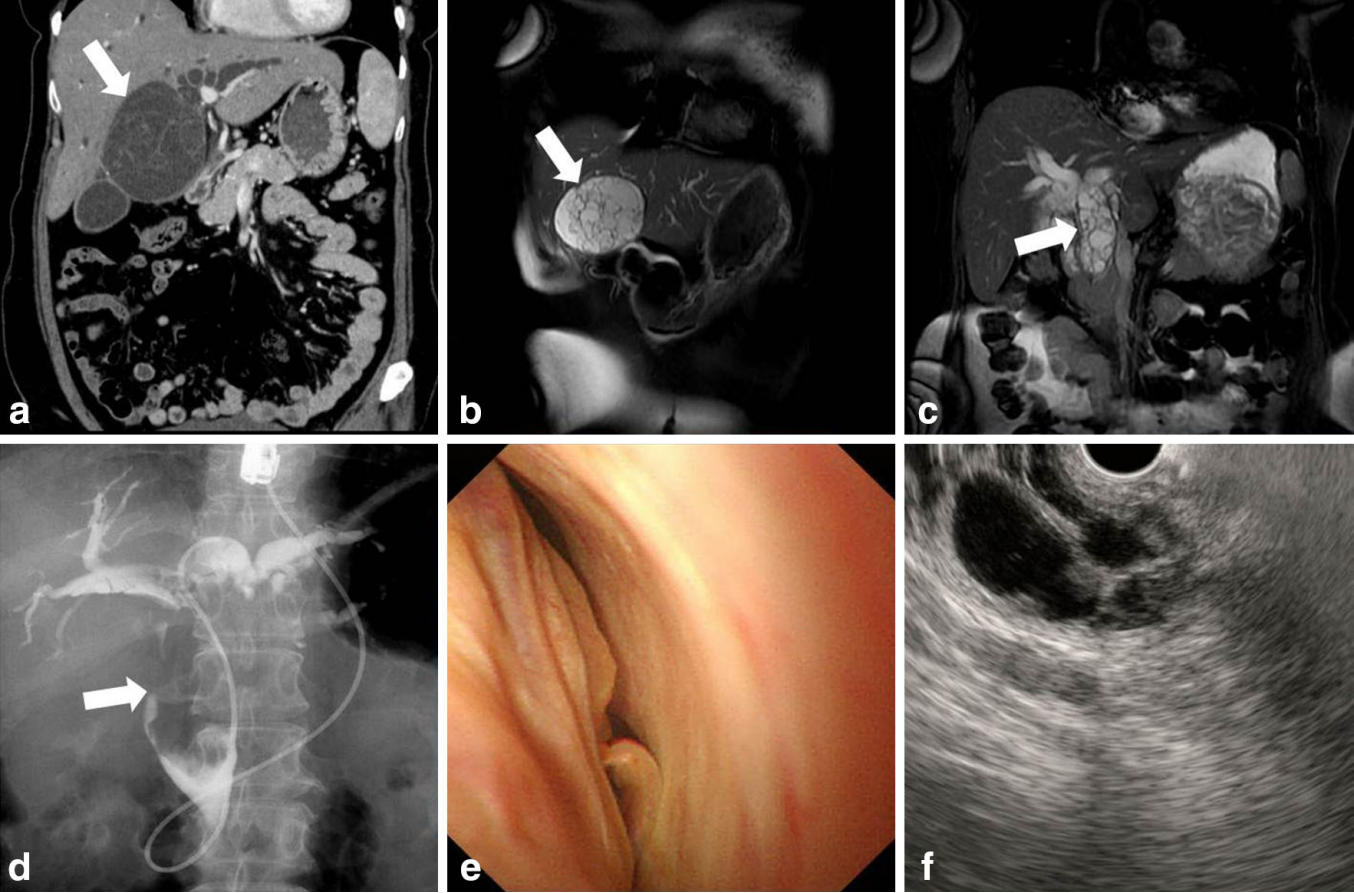
**a** Contrast abdominal CT revealed a multi-locular cystic lesion in the left hepatic lobe (S4), with multiple septal formations (*arrow*). Intrahepatic bile ducts were dilated. **b, c** MRI showed high signal intensities on T2-weighted images of the components inside the cyst (*arrow*). The septal formation was connected to the left hepatic duct and common bile duct (*arrow*), and thus the spread of the tumor into the bile duct was suspected. **d** ERCP revealed an oval-shaped filling defect that appeared to fill the common bile duct (*arrow*). **e** Cholangioscopy enabled a direct observation of the tumor filling the common bile duct. **f** EUS revealed that the tumor occupied the lumen of the common bile duct, and many septal formations were observed

Contrast abdominal computed tomography (CT) revealed a 83 × 80 mm multi-locular cystic lesion with an internal septal formation in the left hepatic lobe (Segment 4: S4) (Fig. 1a). No nodular components, ductal invasion, or distant metastases were found. Magnetic resonance imaging (MRI) showed low signal intensities on T1-weighted images and high signal intensities on T2-weighted images inside the cystic lesion (Fig. 1b). The septal formation was connected to the left hepatic duct and common bile duct, and spread of the tumor to the bile duct was suspected (Fig. 1c). Endoscopic retrograde cholangiopancreatography (ERCP) was performed, and no excretion of mucin from the papilla of Vater was observed. Cholangiography revealed an oval-shaped filling defect in the common bile duct (Fig. 1d). There were no findings indicating malignancy in the bile cytology. Cholangioscopy enabled a direct observation of the smooth tumor wall inside the common bile duct (Fig. 1e). Endoscopic ultrasound (EUS) revealed that the tumor occupied the lumen of the common bile duct, and many septal formations were observed (Fig. 1f). On the basis of the above findings, the patient was diagnosed with benign MCN-L that had prolapsed into the bile duct. The patient underwent a laparoscopy-assisted left lobectomy, a cholecystectomy, and a right hepaticojejunostomy. The patient progressed well following the surgical procedures and was discharged from the hospital 14 days postoperatively.

**Fig. 2:**
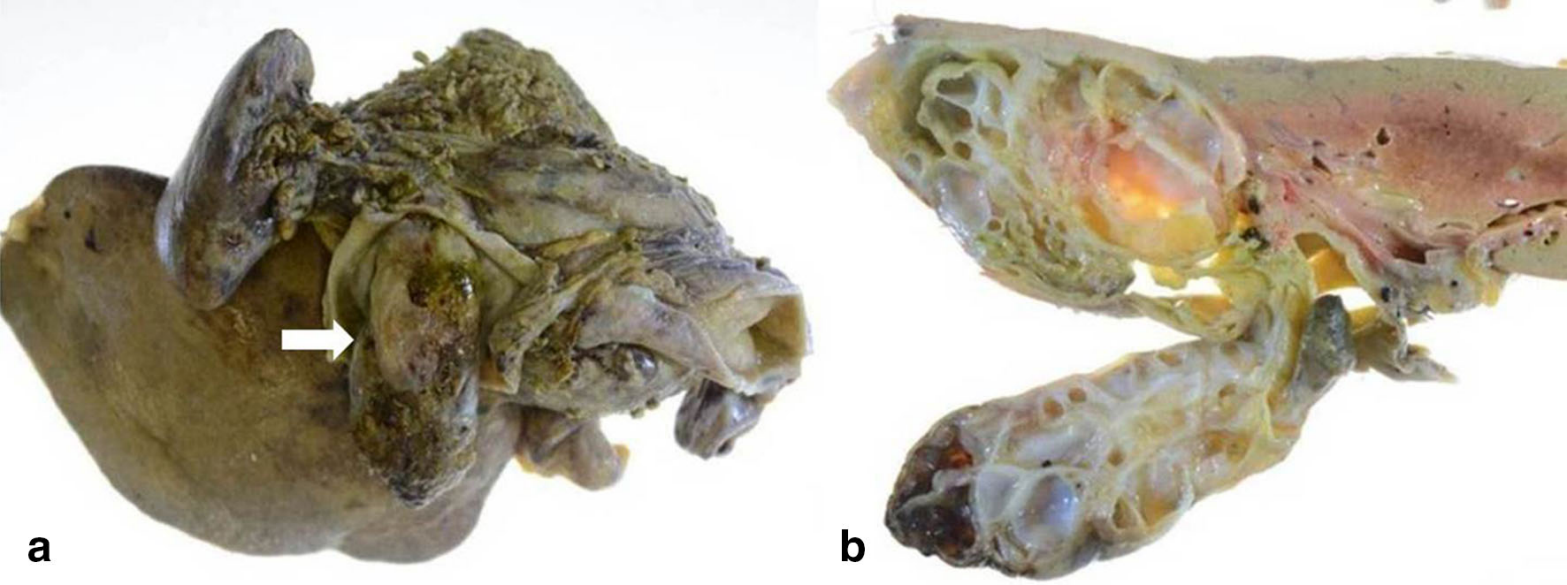
**a** An incision was made in the common bile duct, which was filled with the tumor (*arrow*). **b** A multi-locular cystic tumor was noted in the S4 liver segment, and had prolapsed into the left hepatic duct and common bile duct

**Fig. 3:**
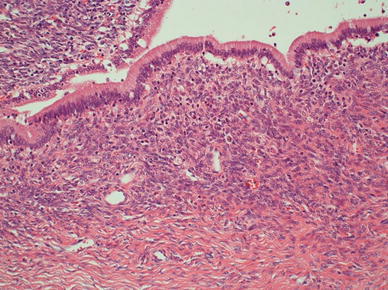
Microscopic findings revealed that the cystic lesion was lined with mucinous cuboidal epithelium, and OLS was observed extensively in the cyst wall stroma. MCN-L with low-grade dysplasia was diagnosed

Macroscopic findings indicated a multi-locular cystic tumor in S4 of the liver. It had prolapsed into the left hepatic duct and common bile duct (Fig. 2a, b). Microscopic findings revealed that the cystic lesion was lined with mucinous cuboidal epithelium, and OLS was observed extensively in the cyst wall stroma (Fig. 3). The OLS was positive for both progesterone and estrogen receptors. A definitive diagnosis of MCN-L with low-grade dysplasia was therefore made.

### Case 2

A 26-year-old woman visited our hospital after being told by a family member that her eyes appeared yellow. Her vital signs were blood pressure 110/72 mmHg, heart rate 68 beats/min, and body temperature 36.2 °C. She presented no tenderness in the abdominal region. Laboratory tests revealed elevated levels of hepato-biliary enzymes (T-bil: 4.1 mg/mL; GOT: 271 U/L; GPT: 394 U/L; ALP: 1568 U/L; γ-GTP: 489 U/L). Tumor marker levels were normal (CA 19-9: 5.5 U/mL; CEA ≦0.5 ng/mL). Both HBsAg and HCVAb were negative and the patient had no history of excessive alcohol consumption.

**Fig. 4:**
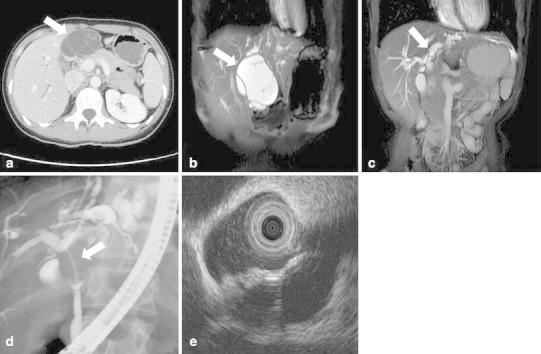
**a** Contrast abdominal CT revealed a cystic lesion with the septal formation in the left hepatic lobe (S4) (*arrow*). **b**, **c** MRI showed high signal intensities on T2-weighted images of the components of the cyst (*arrow*). The septal formations were connected to the left hepatic duct and common bile duct (*arrow*). The spread of the tumor to the bile duct was suspected. **d** ERCP revealed a filling defect in the upper bile duct (*arrow*). The lower tip of the defect area was oval-shaped. Stenosis of the left hepatic duct was also observed. **e** IDUS revealed the spread of the tumor to the common bile duct, with the septal formations inside the tumor

Contrast abdominal CT revealed a 61 × 39 mm multi-locular cystic lesion with internal septal formation in the left hepatic lobe (S4), but no nodular components were contained (Fig. 4a). No ductal invasion or distant metastases were found. MRI showed low signal intensities on T1-weighted images and high signal intensities on T2-weighted images inside the cyst (Fig. 4b). The septal formation was connected to the left hepatic duct and common bile duct, and thus the spread of the tumor to the bile duct was suspected (Fig. 4c). ERCP demonstrated no excretion of mucin from the papilla of Vater. Cholangiography revealed a filling defect in the upper bile duct, and the lower tip of the defect area was oval-shaped. Furthermore, stenosis of the left hepatic duct was observed (Fig. 4d). There were no findings indicating malignancy in the bile cytology. IDUS revealed the spread of the tumor to the left hepatic duct and common bile duct, with a number of septal formations inside the tumor (Fig. 4e). The patient was therefore diagnosed with non-malignant MCN-L and an extensive left lobectomy and right hepaticojejunostomy were performed.

**Fig. 5:**
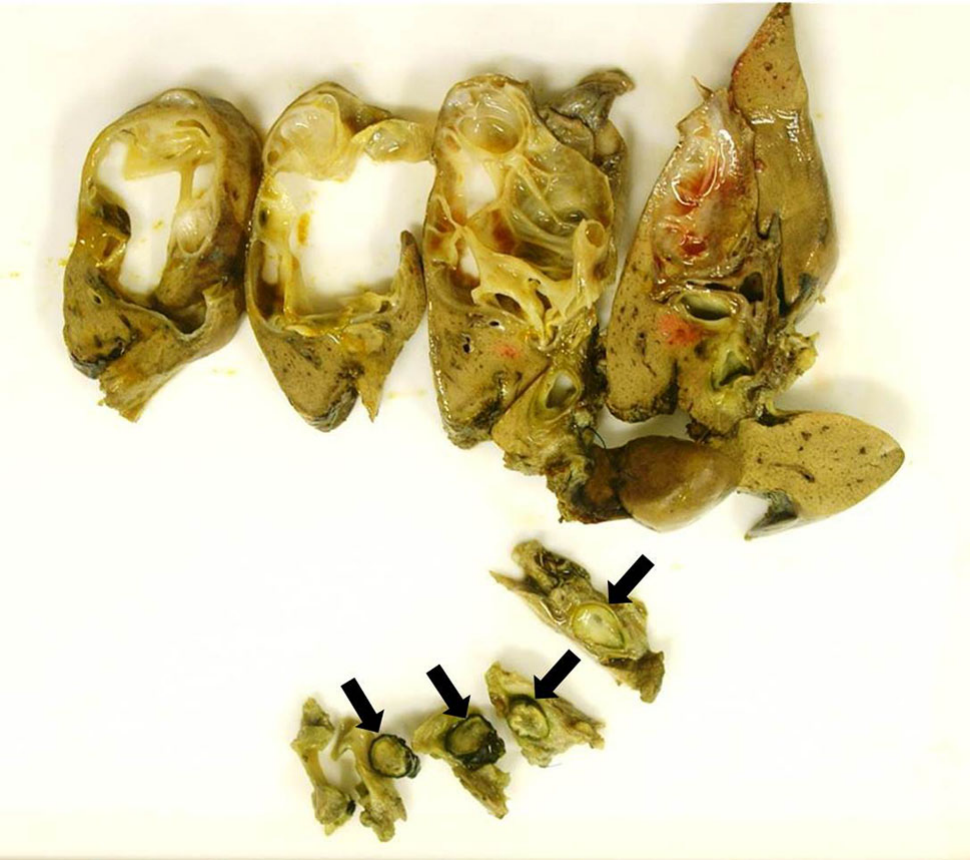
Examination of the resected specimen confirmed that the tumor originated from S4 of the liver. It had prolapsed into the left hepatic duct and common bile duct (*arrow*)

**Fig. 6:**
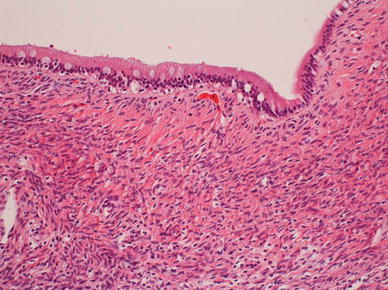
Microscopic findings indicated that the cystic lesion was lined with mucinous cuboidal epithelium, and OLS was observed extensively in the cyst wall stroma. MCN-L with low-grade dysplasia was diagnosed

Examination of the resected specimen confirmed that the tumor originating from S4 of the liver had prolapsed into the left hepatic duct and common bile duct (Fig. 5). Microscopic findings indicated that the cystic lesion was lined with mucinous cuboidal epithelium, and OLS was observed extensively in the cyst wall stroma (Fig. 6). The OLS was positive for both progesterone and estrogen receptors. MCN-L with low-grade dysplasia was diagnosed.

## Discussion

The World Health Organization (WHO) classifies mucus-producing bile duct tumors of the liver into intraductal papillary neoplasm of the bile duct (IPNB) or MCN-L [1]. These concepts were established as counterparts to intraductal papillary mucinous neoplasm (IPMN) of the pancreas and mucinous cystic neoplasm of the pancreas (MCN-P). IPNBs become papillary inside the bile duct and secrete mucin in approximately one-third of all cases. They show communication with the bile duct and do not exhibit OLS. As in the case of IPMN of the pancreas, four phenotypes are known to exist––pancreatobiliary, intestinal, oncocytic, and gastric. On the other hand, MCN-Ls are cyst-forming epithelial tumors that are composed pathologically of mucus-producing cuboidal or columnar epithelium. They usually have no communication with the bile duct and exhibit OLS.

MCN-Ls have been traditionally called biliary cystadenomas or biliary cystadenocarcinomas [2, 3]. The definitions of these tumors have been controversial; however, in recent years, OLS has been considered necessary for a MCN-L diagnosis, and therefore the disease concept is now being established. Thus, tumors that have been referred to as biliary cystadenomas or biliary cystadenocarcinomas in previous reports could be classified into tumors with and without OLS. Most biliary cystic neoplasms that have been reported previously as biliary cystadenomas or cystadenocarcinomas without OLS are currently thought to be IPNBs [2, 4–8]. Based on the 2010 WHO classifications, biliary cystic neoplasm with OLS should be diagnosed as MCN-L.

Zen et al. [9] investigated 54 cases of MCN-L and reported the following characteristics: MCN-Ls often occurred in the left hepatic lobe (72 %), the mean age of onset was 52.5 years, and they rarely developed in men (over 90 % of the patients were women). In addition, MCN-Ls are often large, with a mean size of 100 mm (29–240 mm). There is little correlation with viral hepatitis, intrahepatic cholelithiasis, or liver cirrhosis, and malignancy is uncommon, with 1 out of 54 cases exhibiting micro-invasive carcinoma. Although rare, MCN-Ls have the potential to be malignant. Even for asymptomatic cases, surgery is generally preferable for MCN-L. MCN-Ps, the same type of tumor but located in the pancreas, are known to have relatively low malignant potential, but invasive carcinomas have been reported to account for approximately 10 % of cases [10]. Zen et al. suggested that both MCN-Ls and MCN-Ps exhibit low malignant potential; however, of the two, MCN-Ls exhibit lower malignant potential [9].

Preoperative differentiation of benign and malignant MCN-Ls is difficult. Previous reports of malignant MCN-Ls indicate nodular components with contrast enhancement effects within the cyst, ductal invasion, and metastasis to other organs [2, 11]. In our cases, contrast CT showed no nodules inside the cyst, no ductal invasion, and no metastasis to other organs. There were no findings indicating malignancy in the bile cytology. The above findings suggested the presence of benign MCN-L preoperatively. However, a definitive diagnosis can only be made based on postoperative pathological findings.

Kubota et al. [12] investigated 119 cases of IPNB and 9 cases of MCN-L, all of which occurred in females, and most MCN-L cases were asymptomatic in comparison with the IPNB cases. They suggested that patients with IPNBs may be more likely to develop symptoms such as cholangitis and liver dysfunction due to communication with the bile duct. However, the tumors of the 2 patients in the present report had prolapsed into the bile duct and exhibited expansive growth. Obstructive jaundice was therefore observed.

We searched the literature for MCN-L cases in which the tumor grew expansively in the bile duct over the past 10 years (from 2004 to 2014). In our literature search, we used the keywords “biliary cystadenoma”, “biliary cystadenocarcinoma”, and “mucinous cystic neoplasm of the liver” to search PubMed and extracted cases with growth in the bile duct. We only diagnosed cases with OLS as MCN-Ls and excluded cases that did not exhibit OLS or for which no mention of OLS was made. We also excluded any cases that were complicated by IPNB.

As a result, 15 cases were extracted [13–27] and are listed in Table 1. The mean age of these patients was 44.5 years (25−62 years), all of the patients were female, and all showed the following symptoms: jaundice (9 patients), abdominal pain (9 patients), and liver dysfunction (2 patients); some patients had multiple symptoms. Tumor size was recorded in 11 of the 15 patients. The mean size was 49.5 mm (18−79 mm); the primary tumor sites included the left hepatic lobe (10 patients: 8 cases of S4, 1 case of S3, 1 case of unknown), the common bile duct (3 patients), and the left hepatic duct (2 patients). The tumor spread to both the left hepatic duct and the common bile duct in 7 patients, only the common bile duct in 5 patients, and only the left hepatic duct in 3 patients. ERCP images were recorded for 7 patients. In all of the patients, the images revealed the same characteristic oval-shaped filling defects in the bile duct, as seen in the 2 patients in our study. A hepatectomy and bile duct resection were performed to completely remove the lesions. Pathological examination indicated that none of tumors was malignant.

**Table 1:** Literature cases

Reference number	Age (years)	Sex	Symptoms	Size	Primary site of the tumor	Development of the tumor	ERC image	Operation	Pathological diagnosis
13	62	F	Jaundice	41 mm	Common bile duct	Common bile duct	Oval-shaped filling defect	Bile duct resection	Cystadenoma of the common bile duct
14	34	F	Recurrent episodes of obstructive jaundice	45 mm	Left lobe (S4)	Left hepatic duct, common bile duct	–	Bile duct resection	Biliary cystadenoma
15	41	F	Epigastric pain and obstructive liver enzymes	30 mm	Left lobe (S4)	Left hepatic duct	Oval-shaped filling defect	Left lobectomy, bile duct resection	Hepatobiliary cystadenoma
16	37	F	Abdominal bloating	29 mm	Left lobe (S4)	Left hepatic duct, common bile duct	Oval-shaped filling defect	Left lobectomy, bile duct resection	Biliary cystadenoma
17	39	F	Obstructive jaundice	–	Left lobe (S4)	Left hepatic duct, common bile duct	Oval-shaped filling defect	Left lobectomy, bile duct resection	Biliary mucinous cystadenoma
18	25	F	Hypochondrial pain	55 mm	Left lobe (S4)	Left hepatic duct, common bile duct	–	Left lobectomy	Biliary cystadenoma
19	62	F	Dysuria and hyperpigmentation of urine	–	Left hepatic duct	Common bile duct	–	Extended left lobectomy, bile duct resection	Extrahepatic cystadenoma
20	28	F	Upper abdominal pain	73 mm	Left lobe (S4)	Common bile duct	Oval-shaped filling defect	Segmentectomy (left medial section), bile duct resection, cholecystectomy	Hepatobiliary cystadenoma
21	56	F	Right hypochondrial pain, spontaneously remitted jaundice	55 mm	Left lobe (S4)	Left hepatic duct, common bile duct	–	Left lobectomy	Biliary cystadenoma
22	57	F	Right hypochondrial pain, jaundice	50 mm	Left lobe (S4)	Left hepatic duct	–	Left lobectomy, bile duct resection, cholecystectomy	Hepatobiliary cystadenoma
23	42	F	Jaundice	–	Common bile duct	Common bile duct	Oval-shaped filling defect	Bile duct resection	Biliary cystadenoma
24	54	F	Abdominal pain, abnormal liver function tests	18 mm	Common bile duct	Common bile duct	–	Bile duct resection	Biliary cystadenoma
25	58	F	Hypochondrial pain, jaundice	–	Left hepatic duct, common bile duct	Left hepatic duct, common bile duct	Oval-shaped filling defect	Bile duct resection	Biliary cystadenoma
26	40	F	Epigastric pain, jaundice	70 mm	Left lobe	Left hepatic duct	–	Left lobectomy	Biliary cystadenoma
27	32	F	Abdominal pain, jaundice	79 mm	Left lobe (S3)	Left hepatic duct, common bile duct	–	Left lobectomy	Biliary cystadenoma

The MCN-Ls that spread to and grew in the bile duct developed in patients who were younger than those described in the report by Zen et al., and the tumors tended to be smaller. This may have been because the spread of the tumors to the bile duct is more likely to cause symptoms, such as jaundice or cholangitis, leading to early detection. The characteristic oval-shaped filling defects that are observed with ERCP may be extremely useful in diagnosing this tumor. The most common primary tumor site was S4 of the liver. The primary tumor site was also S4 of the liver in the 2 patients in our reports, suggesting that MCN-Ls that develop from this site may be prone to prolapse into the bile duct. This may be because the most common primary site of MCN-L is S4 of the liver [2], but it is potentially an interesting phenomenon. We hypothesize that because S4 of the liver is near the hepatic portal region and close to the relatively thick central side of the bile duct, the tumors originating from this site may be prone to prolapse into the bile duct. MCN-Ls in the 15 cases that we reviewed were all benign. Prolapse into the bile duct may be a finding which suggests that the tumor is benign. However, as malignant MCN-Ls are relatively uncommon, we have not reviewed enough cases to date to make such conclusions.

In addition, we will discuss bile duct lesions of other liver tumors and compare them with MCN-L. Bile duct invasion of hepatocellular carcinomas (HCCs) is clinically rare, but reports suggest that it has been observed in 5 % of HCCs in autopsies [28]. Such cases exhibit irregular stenosis and exclusion of the bile duct. There are three main categories of cholangiocellular carcinoma (CCC): mass-forming (MF), periductal infiltrative (IF), and intraductal growth (IG). Of these, the IG type overlaps the IPNB concept proposed in recent years, with irregular papillary tumors developing in the bile duct. The frequency of the IG type is reported to be 4 % of CCC cases [29]. Papillary tumors are confirmed on cholangiography and cholangioscopy. Growth of other tumor-related lesions into the bile duct (focal nodular hyperplasia, hepatic adenoma, lymphoma, hepatoblastoma, and mesenchymal tumor) is exceptionally rare.

The occurrence of MCN-Ls prolapsing into the bile duct is unclear as it is an extremely rare tumor. Prolapse into the bile duct has a strong impact, making it likely to be reported. Examination of further cases is required. In MCN-L, expansive growth is shown by prolapse into the bile duct so that the smooth, round tumor occupies the lumen of the bile duct. This development of the tumor, which is completely different from HCC invasion into the bile duct and the IG type of CCC, can be said to be a characteristic behavior of MCN-L.

MCN-L is basically considered to be a non-invasive, benign tumor that does not grow invasively but grows expansively. This tumor can prolapse into the bile duct and then grow to fill the bile duct. ERCP can be used to confirm the characteristic oval-shaped filling defect in the bile duct. Even if the entire oval shape is not revealed, as in case 2, careful attention should be paid to the fact that the margins of the filling defect are oval. In addition, cholangioscopy, IDUS, and EUS are useful for differentiating these tumors from stones.

We described 2 MCN-L cases that exhibited extremely interesting spread patterns. Prolapse into the bile duct and expansive growth may be a characteristic behavior of MCN-L. Additional data from more cases needs to be collected for further elucidation of MCN-L’s pathophysiology.

## Abbreviations

MCN-L: Mucinous cystic neoplasm of the liver
S4 of the liver: Segment 4 of the liver
ERCP: Endoscopic retrograde cholangiopancreatography
EUS: Endoscopic ultrasound
IDUS: Intraductal ultrasound
OLS: Ovarian-like stroma
CA 19-9: Cancer antigen 19-9
CEA: Carcinoembryonic antigen
HBsAg: Hepatitis B surface antigen
HCVAb: Hepatitis C virus antibody
CT: Computed tomography
MRI: Magnetic resonance imaging
WHO: World Health Organization
IPNB: Intraductal papillary neoplasm of the bile duct
IPMN: Intraductal papillary mucinous neoplasm
MCN-P: Mucinous cystic neoplasm of the pancreas
HCC: Hepatocellular carcinoma
CCC: Cholangiocellular carcinoma
